# Protective Effects of *Hericium erinaceus* Mycelium and Its Isolated Erinacine A against Ischemia-Injury-Induced Neuronal Cell Death via the Inhibition of iNOS/p38 MAPK and Nitrotyrosine

**DOI:** 10.3390/ijms150915073

**Published:** 2014-08-27

**Authors:** Kam-Fai Lee, Jiann-Hwa Chen, Chih-Chuan Teng, Chien-Heng Shen, Meng-Chiao Hsieh, Chien-Chang Lu, Ko-Chao Lee, Li-Ya Lee, Wan-Ping Chen, Chin-Chu Chen, Wen-Shih Huang, Hsing-Chun Kuo

**Affiliations:** 1 Department of Pathology, Chang Gung Memorial Hospital, Chiayi 61363, Taiwan; E-Mail: lkf2002@adm.cgmh.org.tw; 2 Institute of Traditional Medicine, School of Medicine, National Yang-Ming University, Taipei 112, Taiwan; E-Mail: chenjiannhwa@yahoo.com.tw; 3 School of Medicine, Fu-Jen Catholic University, Taipei 24205, Taiwan; 4 Department of Emergency Medicine, Cathay General Hospital, Taipei 22174, Taiwan; 5 Department of Nursing and Chronic Diseases and Health Promotion Research Center, Chang Gung University of Science and Technology, Chiayi 61363, Taiwan; E-Mail: ccteng5648@gmail.com; 6 Department of Hepato-Gastroenterology, Chang Gung Memorial Hospital, Chiayi 61363, Taiwan; E-Mail: gi2216@adm.cgmh.org.tw; 7 Division of Colon and Rectal Surgery, Department of Surgery, Chang Gung Memorial Hospital, Chiayi 61363, Taiwan; E-Mail: mr8872@gmail.com; 8 Graduate Institute of Clinical Medical Sciences, College of Medicine, Chang Gung University, Taoyuan 330, Taiwan; 9 Department of Colorectal Surgery and Department of Surgery, Chang Gung Memorial Hospital, Kaohsiung 833, Taiwan; E-Mails: cclu999@gmail.com (C.-C.L.); kmch4329@gmail.com (K.-C.L.); 10 Kaohsiung Medical Center, Chang Gung University College of Medicine, Kaohsiung 833, Taiwan; 11 Grape King Biotechnology Inc., Zhong-Li 320, Taiwan; E-Mails: ly.lee@grapeking.com.tw (L.-Y.L.); wp.chen@grapeking.com.tw (W.-P.C.); gkbioeng@grapeking.com.tw (C.-C.C.); 12 Department of Nursing, Chang Gung University of Science and Technology, Chiayi 61363, Taiwan; 13 Chronic Diseases and Health Promotion Research Center, Chang Gung University of Science and Technology, Chiayi 61363, Taiwan; 14 Research Center for Industry of Human Ecology, Chang Gung University of Science and Technology, Taoyuan 333, Taiwan

**Keywords:** *Hericium erinaceus*, neuroprotection, erinacine A, ischemia reperfusion, iNOS

## Abstract

*Hericium erinaceus*, an edible mushroom, has been demonstrated to potentiate the effects of numerous biological activities. The aim of this study was to investigate whether *H. erinaceus* mycelium could act as an anti-inflammatory agent to bring about neuroprotection using a model of global ischemic stroke and the mechanisms involved. Rats were treated with *H. erinaceus* mycelium and its isolated diterpenoid derivative, erinacine A, after ischemia reperfusion brain injuries caused by the occlusion of the two common carotid arteries. The production of inflammatory cytokines in serum and the infracted volume of the brain were measured. The proteins from the stroke animal model (SAM) were evaluated to determine the effect of *H. erinaceus* mycelium. *H. erinaceus* mycelium reduced the total infarcted volumes by 22% and 44% at a concentration of 50 and 300 mg/kg, respectively, compared to the SAM group. The levels of acute inflammatory cytokines, including interleukin-1β, interleukin-6 and tumor necrosis factor á, were all reduced by erinacine A. Levels of nitrotyrosine-containing proteins, phosphorylation of p38 MAPK and CCAAT enhancer-binding protein (C/EBP) and homologous protein (CHOP) expression were attenuated by erinacine A. Moreover, the modulation of ischemia injury factors present in the SAM model by erinacine A seemed to result in the suppression of reactive nitrogen species and the downregulation of inducible NO synthase (iNOS), p38 MAPK and CHOP. These findings confirm the nerve-growth properties of Hericium erinaceus mycelium, which include the prevention of ischemic injury to neurons; this protective effect seems to be involved in the *in vivo* activity of iNOS, p38 MAPK and CHOP.

## 1. Introduction

*Hericium erinaceus* (Lion’s mane or Yamabushitake) is a mushroom that grows on old or dead broadleaf trees. *H. erinaceus* is consumed as a food in Japan and China without harmful effects. *H. erinaceus* has been extensively documented and possesses a range of therapeutic properties such as antioxidant activity [1], hypolipidemic activity [2], hemagglutinating activity [3], anti-microbial activity [4] anti-tumorigenic activity [5] and endoplasmic reticulum (ER) stress-modulated activity [6,7]. Erinacines (A–I), the major active agent isolated from the cultured mycelia of *H. erinaceus*, are diterpenoid compounds that exert biological properties through a varied stimulation of nerve growth factor (NGF) synthesis *in vitro* in astrocytes [8,9,10]. It is important to note that oral administration of erinacine A seems to prevent age-related dementia, increasing the level of NGF in the locus coeruleus and hippocampus, but not in the cerebral cortex in rats [11,12]. However, it has not been reported that the *H. erinaceus* mycelium contains erinacines and the mechanism by which erinacine A initiates neuroprotection against ischemic injury to the brain remains poorly understood.

Stroke is the fourth-leading cause of death and the primary cause of long-term disability worldwide [13]. Ischemic insult produces excessive free radicals, which then cause abnormal endoplasmic reticulum (ER) stress signaling that has been linked to apoptosis; these events then induce apoptotic cell death in the neurons [14]. Further changes in stroke, including neurotoxicity mediated by free radicals, also occur; this finding is supported by the fact that ischemic injury is reduced following the induction of endogenous antioxidant pathways and by the presence of scavengers targeting nitric oxide [15]. Thus, a fundamental role for free radical scavengers is recognized for potential neuroprotective agents. This is because ischemic injury is caused by a series of events involving energy depletion and cell death, and these are mediated by various intermediate factors including an excess of extracellular excitatory free-radical formation and the presence of inflammation [16]. After global cerebral ischemia with arterial occlusion, necrotic cell death occurs predominantly in the ischemic core [13]. Reduced blood flow resulting from arterial occlusion or hypotension leads to tissue hypoxia and hypoglycemia, which then cause protein misfolding and endoplasmic reticulum stress. Ischemia-reperfusion trauma of the brain then induces oxidative stress, which leads to the production of nitric oxide (NO), a mediator of protein nitrosylation, and other reactive oxygen species (ROS) [17]. Therefore, it is imperative to develop more effective drugs from natural compounds of edible mushrooms to avoid neuronal death in the penumbral region of the ischemic injury.

Reactive oxygen species derived from ischemia-reperfusion have been shown to be associated with the ER stress-signaling pathway that leads to neuronal survival or death [18]. After an ischemic stroke, iNOS/NO is expressed and participates in the late phase of tissue damage, thereby generating reactive nitrogen species (RNS) and contributing to oxidative stress, which has been extensively studied. The p38 MAPK/CHOP pathway promotes cellular death involved in ER stress-induced apoptosis in the neurons [19]. While considering the key role of iNOS/RNS and p38 MAPK/CHOP in cell death using a stroke model of ischemia reperfusion brain injury, in the present study we shall determine whether *H. erinaceus* is able to effectively improve the neuroprotective effects.

**Figure 1:**
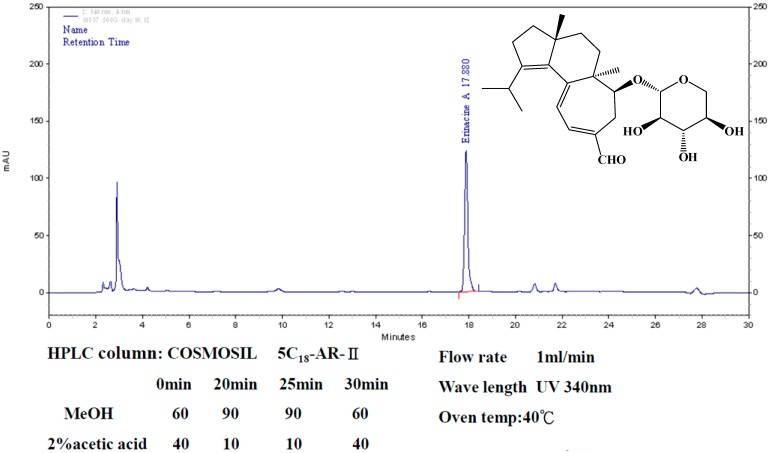
HPLC analysis of erinacine A. The HPLC chromatograms of erinaince A standard sample (**right**) and the ethanol extract of *H. erinaceus* mycelium (**bottom**) from 20-ton bioreactor (UV detection at 340 nm). Retention time of erinacine A is 17.4 min.

This paper focuses on exploring the biological agent of erinacine A and its effect on ischemia reperfusion injury. Fractionation from *H. erinaceus* mycelium and its structural elucidation by ethanol extraction and HPLC analysis techniques were used in Figure 1 [20]. In this study, a cerebral global ischemic stroke model of temporary ischemia followed by reperfusion onset was used to assess whether *H. erinaceus* has a neuroprotective effect. In conclusion, *H. erinaceus* contains an active compound that inhibits global cerebral ischemic injury via inactivation of the iNOS/RNS and p38 MAPK/CHOP pathway; this compound is erinacine.

## 2. Results

### 2.1. Effects of Hericium erinaceus Mycelium on the Suppression of Brain Damage in Ischemic Rats

**Figure 2:**
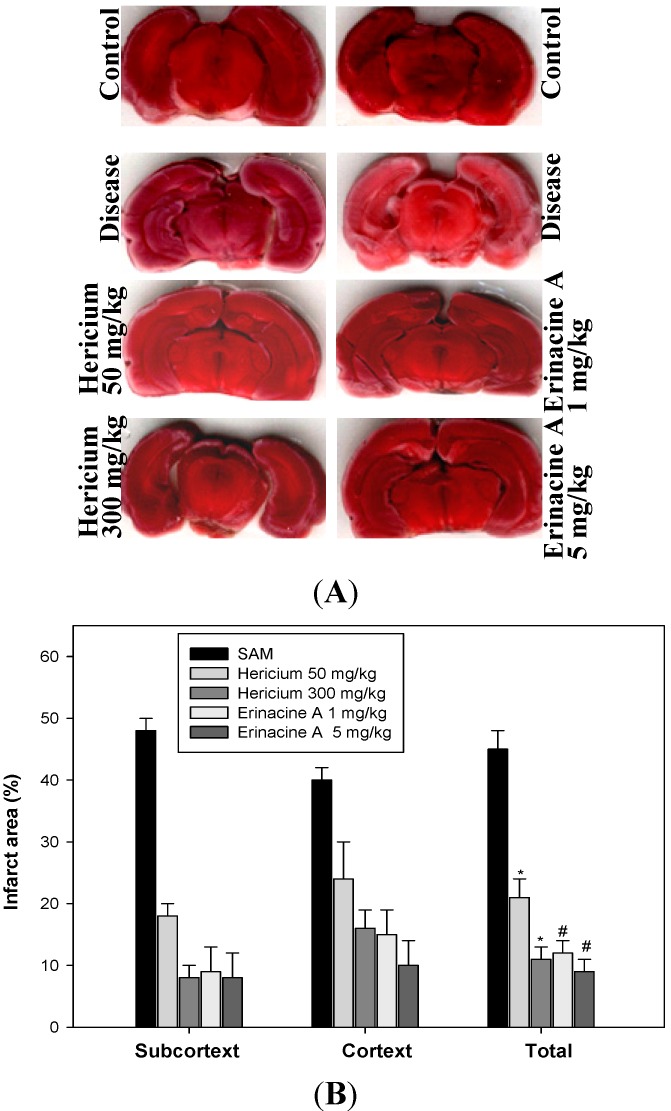
Effects of *Hericium erinaceus* mycelium on infarcted volume in ischemic rats. The stroke animal model (SAM rat 1.5 h and reperfusion 24 h) was applied to rats as described in Experimental Section 4.2. *H. erinaceus* and erinacine A were administered intraperitoneally 90 min after the initial ischemia. The control group and SAM group animals received an equal volume of solvent. (**A**) Representative ischemic lesions at 2 mm thick coronal section from *H. erinaceus* at the dose of 50 and 300 mg/kg and erinacine A at the dose of 1 and 5 mg/kg (injection at 90 min in pre-stroke rats) as well as DMSO-treated rats at 1 day 2,3,5-triphenyl tetrazolium chloride (TTC) staining before ischemia; (**B**) Quantitative analysis of total infarcted volume to determine the therapeutic dose in groups of six rats treated with DMSO (control) or *H. erinaceus* and erinacine A at 24 h in pre-stroke rats. Cortex, subcortex, and total infarcted volumes were determined in the transient stroke rats (shown as percentage of the hemisphere). Data are expressed as mean ± SD of independent experiments. *n* = 6. * *p* < 0.05, SAM group *versus* SAM + *H. erinaceus* group. # *p* < 0.05, SAM group *versus* SAM + erinacine A group.

To determine whether *H. erinaceus* mycelium and its isolated compund erinacine A provide tangible therapeutic benefits by suppressing brain impairment in a rat model that resembles ischemic stroke, we exposed ischemia reperfusion rats to a range of *H. erinaceus* doses for 5 days and performed 2,3,5-triphenyl tetrazolium chloride (TTC) staining assays. As shown in Figure 2, oral administration of *H. erinaceus* had a protective effect on brain infarction size compared to a solvent control. *H. erinaceus* was able to significantly reduce the total infarction size in the brains of SAM rats (* *p* < 0.05, *n* = 6). In addition, stroke-induced infarcted volume was abolished by an erinacine A indicated dose, rather than in the untreated control. Significant differences between the untreated and *H. erinaceus*-treated SAM groups were found for both cortical and subcortical infarctions.

### 2.2. Hericium erinaceus Mycelium Inhibits Neuronal Cell Death in a Transient Stroke Animal Model

**Figure 3:**
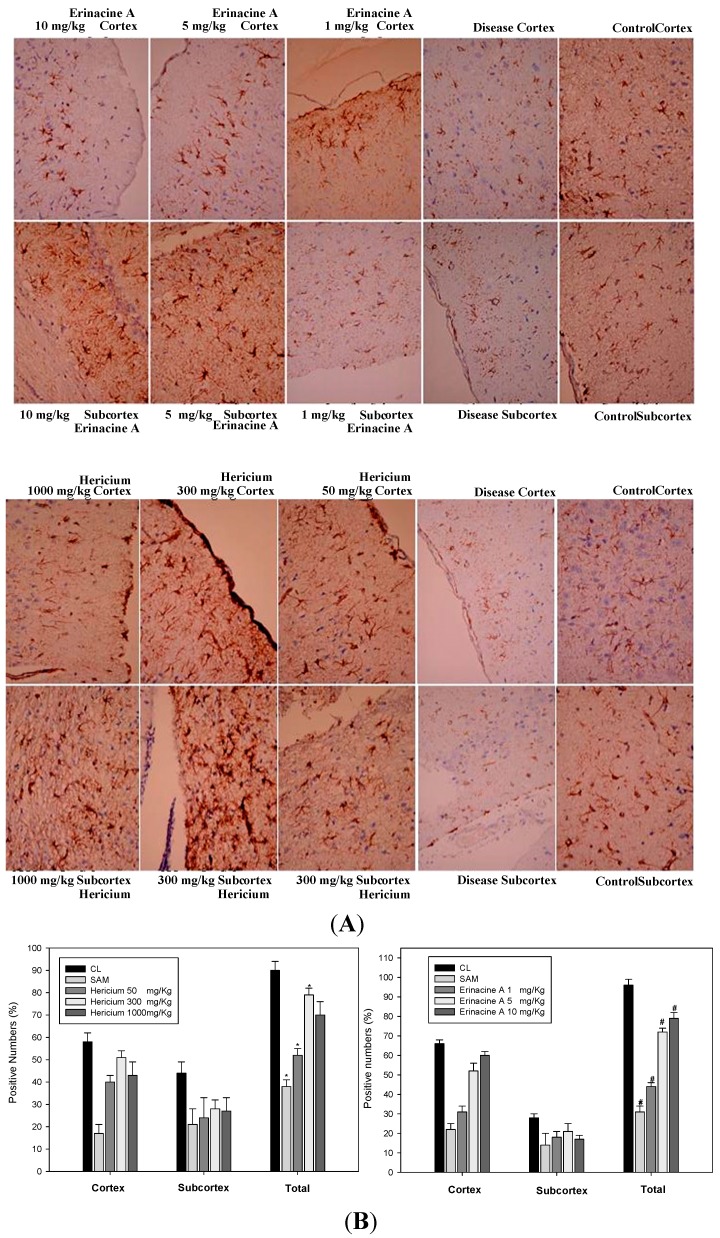
Effect of *Hericium erinaceus* mycelium (HEM) on brain histological examination in transient stroke animal model. Rats were treated without (control group, CL) or with *H. erinaceus* mycelium by oral HEM administration (50, 300, 1000 mg/kg) and i.p. erinacine A injection (1, 5, 10 mg/kg). Histological examination of the brain revealed cortex and subcortex zones as indicated by immunohistochemical staining of NeuN. (**A**) Rats were sacrificed and the brains were separated. Representative brain sections stained as: the control group; rats with transient SAM; HEM treatment (50, 300, 1000 mg/kg) staining of brain tissue. Erinacine A treatment (1, 5, 10 mg/kg) staining; (**B**) Evaluation of pathological examination and NeuN expression were quantitative in brain tissue. The cells were counted from 10 fields (200× magnification) of each brain sample. The results from statistical analysis are the means of cells and were calculated per microscope field from six animals per group. Data are expressed as the mean ± SD of independent experiments. *n* = 6. * *p* < 0.05, SAM group *versus* SAM + HEM group. # *p* < 0.05, SAM group *versus* SAM + erinacine A group.

Neuron-specific protein NeuN is expressed exclusively in most neuronal cell types of vertebrates. Figure 3A shows the expression of NeuN in both cortical and subcortical infarctions of rats. Global immunoreactivity of NeuN was found to occur mostly in the total brain area in SAM rats 24 h after oral administration of *H. erinaceus* and erinacine A intraperitoneally. Quantification of the neuronal cells in terms of pathology showed that *H. erinaceus* mycelium increases the number of normal neurons by 15% and 45% at concentrations of 50 and 300 mg/kg, respectively, compared to the untreated SAM group for both cortical and subcortical infarctions (see Figure 3B, * *p* < 0.05). There was also an increase in the number of positive NeuN cells, when the SAM rats treated with 1, 5 and 10 mg/kg erinacine A were compared with the untreated SAM rats (Figure 3B, *#* *p* < 0.05).

### 2.3. Effects of H. erinaceus Mycelium on Serum Protein ROS Oxidants and Acute Inflammation Cytokines

**Figure 4:**
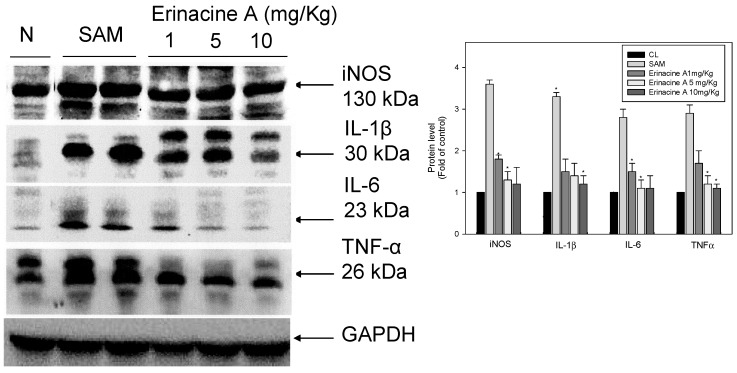
*Hericium erinaceus* mycelium erinacine A-mediated serum protein expression of acute inflammation cytokines in transient stroke animal model. Rat serum with SAM infusion had high levels of oxidative stress and inflammation. Equal amounts of serum for the indicated treatments were analyzed by SDS-PAGE and subsequently immunoblotted with antisera against iNOS, IL-1, IL-6 and TNF, with GAPDH serving as an internal control. Protein levels were quantified by densitometric analysis with the control being set at 100%. Data are presented as the mean ± SD of 3 independent experiments. * *p* < 0.05, when compared with the SAM group.

Recent studies have shown evidence of involvement of ischemia/reperfusion-related stroke disease in the induction of excessive free radicals after brain tissue damage. These have been reported to be highly up-regulated in response to increased levels of pro-inflammatory cytokines such as IL-1β, IL-6, and TNF-α [21]. In our study, the administration of erinacine A to SAM rats resulted in a significant decrease in the secretion of various serum proteins. Moreover, the dose of magnolol (5 mg/kg) resulted in considerably greater inhibition of iNOS expression and IL-1β/IL-6/TNF-α secretion compared to the untreated SAM group (Figure 4).

### 2.4. Immunohistochemistry Stain of iNOS and Nitrotyrosine Proteins on Brain Histopathology by Erinacine A in Transient Stroke as Seen in Animal Model Rats

**Figure 5:**
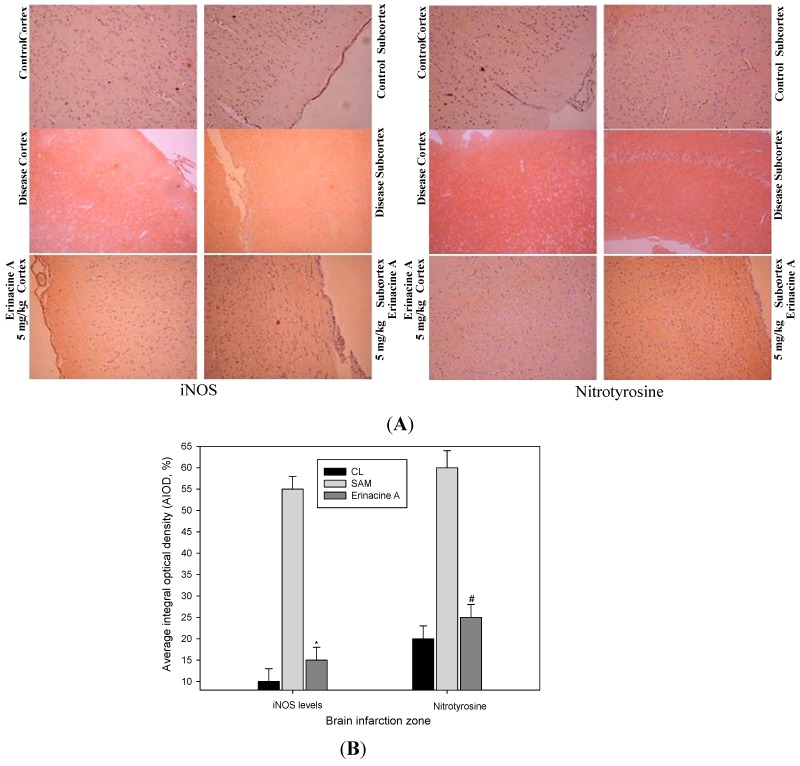
Effect of erinacine A on brain histological iNOS and nitrotyrosine protein expression in a transient stroke animal model. Rats were treated as described. Histological examination of the brain revealed the cortex and subcortex zones as indicated by immunohistochemical staining. (**A**) Rats were sacrificed and the brains were separated. Results show representative brain sections stained for the control group (CL); rats with transient SAM; Erinacine A treatment (10 mg/kg) staining of brain tissue; (**B**) Evaluations of nuclear protein expression and iNOS and nitrotyrosine expression were quantitative in the cortex zone and subcortex zone. The positive stained area was evaluated from three randomly selected observation fields of each brain cortex and subcortex section. Quantitative immunohistochemical proteins were evaluated by average integrated optical density (AIOD). The positive stained area was evaluated from three randomly selected observation fields of each liver section. Data were expressed as the mean ± SD (*n* = 6/group). *, # *p* < 0.05, compared with the control group.

This study demonstrate that erinacine A was able to inhibit inflammatory cytokine expression, which suggests that the brain tissue trauma effectors of nitrotyrosine (RNS) via iNOS/p38/MAPK/CHOP may contribute to the neuroprotective effects of erinacine A. To confirm the effects of *H. erinaceus* mycelium on the therapeutic evaluation, we employed immunohistochemistry to examine these proteins on brain infarctions of the control group, the SAM group and the erinacine A groups. As Figure 5 shows, erinacine A regimens significantly demonstrated a reduced expression of iNOS and nitrotyrosine (RNS).

### 2.5. Effects of Physiological Parameters Indicated by H. erinaceus Mycelium in a Transient Stroke Animal Model

**Figure 6:**
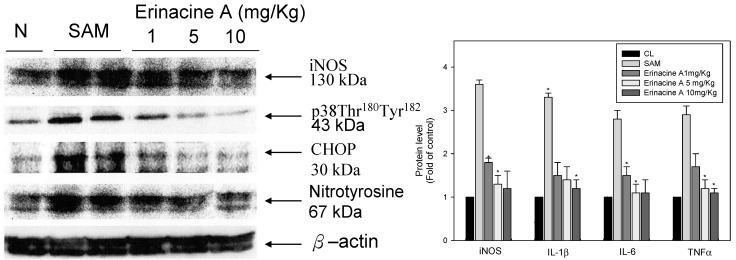
Western blot analysis of iNOS, p38 MAPK and CHOP immunoreactivity after transient global ischemia. Western blot analysis of iNOS, phosphorylation of p38 MAPK, CHOP and nitrotyrosine protein expression in transient stroke as seen in animal model rats. Mice were treated with (control group, CL) or without erinacine A. Representative brain control liver; rats with transient global ischemia; rats that received magnolol treatment (1, 5, 10 mg/kg). Equal amounts of protein from total cell lysates of the rat brain treated with magnolol were analyzed using 10% SDS-PAGE and subsequently treated with antibodies against iNOS, p38 MAPK, CHOP, and nitrotyrosine. â-Actin served as the internal control. Protein levels were quantified by densitometric analysis with the control being set at 100%. Data are presented as the mean ± SD of 3 independent experiments. * *p* < 0.05, when compared with the control group.

Moreover, as Figure 6 shows, erinacine A treatment was found to result in a remarkable inhibition in iNOS, p38/MAPK pathway activation as well as in the expression of CHOP, nitrotyrosine protein, compared to the untreated SAM group in rat brain tissue. In rats treated with *H. erinaceus*, we found less CO_2_ elevation in the blood gas leading to mild acidosis at 90 min SAM. The injection of erinacine A in SAM rats produced a dose-dependent increase in PO_2_. Furthermore, the blood glucose was significantly lower in all *H. erinaceus*-treated SAM rats, and in contrast to the SAM group, the decrease was dose dependent (data not shown).

## 3. Discussion

The mechanisms underlying the protective ischemic injury effects of the mushroom components of *H. erinaceus* remain elusive. It has been previously reported that a number of erinacine derivatives from the cultured mycelia of *H. erinaceus*, such as erinacine A, which has been shown to be a mycelium-growth-associated metabolite, stimulate NGF synthesis in cultured astrocytes. NGF induction then correlates with increased nerve cell growth and neurite outgrowth and expands catecholamine in the brain of rats [22,23]. Therefore, it is clinically relevant to search novel chemotherapeutic agents to prevent stroke-induced brain injury. *H. erinaceus* fractions were obtained from the mycelium. Erinacine A composition was 3 mg/g (0.3%) as shown in Figure 1 [20]. In the same study, our results clearly showed that *H. erinaceus* mycelium inhibited neurological insults in ischemic stroke rats (Figure 2). The *in vivo* inhibition effect and mechanism of the action of erinacine A, a diterpenoid derivative (Figure 2), on rat global ischemic stroke for experimental treatments were investigated. In summary, our study demonstrated that the effect of erinacine A as a scavenger of reactive oxygen species inhibits activation of iNOS/p38 MAPK and CHOP protein, which protects neurons from death caused by ischemic injury. Several lines of evidence indicate that the effect of erinacine A seems to have another role in that it affects iNOS expression and the associated pathways; these are involved in neuronal survival/plasticity after ischemia reperfusion injury (Figure 4).

Ischemic damage causes a variety of cellular and molecular responses in the brain when the continuity of oxygen and glucose delivery via blood flow in the brain is interrupted [24]. Such an interruption of the flow can cause the brain to damage neuron cells, and glial expression of inducible nitric oxide (NO) synthase (iNOS) and neuronal death are important [25,26]. Excessive free radicals and massive inflammation resulted in neuronal death via recruited leukocytes and activated microglial cells [27]. Most studies targeting ischemic-related injury after a stroke have involved acute inflammation and have shown that oxidative stress that accompanies the early stages of stroke can result in the activation of transcription factors (e.g., nuclear factor CHOP). They have also revealed increased sensitivity to brain ischemia, suggesting a relative role for this ER stress inducer in stroke damage to disrupt the blood brain barrier (BBB), a brain barrier providing the regulation of homeostasis [28]. Moreover, the CHOP protein in brain ischemic damage regulated by various mechanisms, including the p38 MAPK pathway, which induces ER stress in neurons, activates the unfolded protein response and finally leads to neuronal apoptosis such as stroke [22,29]. As illustrated in Figure 3, these results in our experiment showed that ischemia affected the neuron cells, resulting in the endogenous inducible iNOS and nitrotyrosine (RNS) increasing after the induction of ischemic damage of the brain while treatment with erinacine A could effectively inhibit iNOS/p38 MAPK and CHOP protein expression as a negative regulator of a stroke. As shown in Figure 6, our results demonstrate that erinacine A with three different dosages caused the inhibition of rat neuronal damage by inactivating the iNOS/p38 MAPK-dependent CHOP expression. However, there is less understanding of the mechanisms involved in the suppression of endoplasmic reticulum stress and reactive nitrogen stress, thus increasing neuron-protection and reducing the changes associated with a stroke [30,31]. Further understanding of how *H. erinaceus* mycelium fractions and erinacine A affect the underlying events during acute neuronal insult will clarify whether inhibition of ER stress may help in the development of new practical therapies for the treatment of stroke.

Many earlier reports showed that ischemic brain injury was related to an initiation of neutrophil accumulation in the brain together with the transmigration of adhesion molecules within the brain that induced brain cytokines (TNF-α, IL-1ß and IL-6) and increased the expression of iNOS in the glia; these events occurred in a variety of activated endothelial cells, microglia, neurons, platelets, monocytes, macrophages and fibroblasts [32]. In our study, we proposed the molecular mechanisms underlying the roles of *H. erinaceus* mycelium in controlling inflammatory cytokines during ischemic injury. We also showed that various pro-inflammatory cytokines, including nitrotyrosine, iNOS, IL-1β, TNF-α and IL-6, are up-regulated by artery ischemic reperfusion in rats. Significantly, erinacine A reduces the production of the above cytokines through dose-dependent inhibition (Figure 4).

It is currently known that hypoglycemic patients with cerebral ischemia have severe lactic acidosis and poor clinical outcomes that lead to major metabolic disorders and an increase in brain tissue damage [33,34]. Based on this, we compared the anti-hyperglycemia properties of *H. erinaceus* in stroke animals to further examine the neuroprotection relationships associated with less blood glucose being the major factor in prognosis [33]. Further studies of the administration of *H. erinaceus* mycelium *in vivo* with the addition of glucose whether infarcted volume in *H. erinaceus*-treated SAM rats are still required to validate these findings.

## 4. Experimental Section

### 4.1. Hericium erinaceus Extracts and Analysis of Erinacine A

Fresh mycelium of *H. erinaceus* was extracted with ethanol. The extract was concentrated and fractionated by solvent partition between ethylacetate and water. The ethylacetate fraction was subjected to silica gel column chromatography using *n*-Hexane–ethylacetate as the eluent. The Hexane–acetone eluate was subjected to silica gel column chromatography according to the previous study [20,35]; HPLC analysis of erinacine A was executed according to the previous study with minor modifications. The analytical column used was a COSMOSIL 5C18-AR-II (250 × 4.6 mm; particle size 5 μm, Nacalai USA, Inc., Kyoto, Japan). Separation was performed at 40 °C using two different gradients for the mobile phase, which consisted of two solvents, methanol (A) and 2.0% acetic acid in water (B). The gradient elution had the following profile: 0–20 min, 60%–90% (A); 20–25 min, 90% (A). The retention time of erinacine A was approximately ~17 min at a flow rate of 1.0 mL/min with a scanning UV wavelength at 340 nm. The 3 mg/g erinacine A in the *H. erinaceus* extracted with 85% ethanol was confirmed and quantified by HPLC as shown in Figure 1.

### 4.2. Induction Ischemia Reperfusion Brain Injury and Drug Administration

Adult male Sprague–Dawley rats weighing around 280 ± 20 g were kept individually in a 12-h light/dark cycle cage with free access to water and food. Animal care and the general protocols for animal use were approved by the Institutional Animal Care and Use Committee of Chang Gung University of Science and Technology (2012-003, Affidavit of Approval of Animal Use Protocol). All efforts were made to minimize the number of animals used and their suffering. These rats were operated on according to the modified global cerebral ischemia model, which can be induced by the occlusion of the two common carotid arteries, *i.e.*, by two-vessel occlusion (2VO) to induce reversible ischemia for a limited time period [36]. In brief, the clamping of the bilateral carotid arteries was ligated with 4-0 nylon under anesthetic using 10% chloral hydrate (350 mg/kg). These filaments were withdrawn 90 min after the onset of ischemia. The femoral artery and vein were exposed and cannulated with PE-50 polyethylene tubing (Fisher Scientific, Pittsburgh, PA, USA) [36]. The arterial catheter was used for continuous blood pressure recording and blood gas analysis (AVL 990; Homburg, Germany). Body temperature was maintained at 37 °C via a homoeothermic blanket. Sham-operated control animals underwent all the surgical procedures, except the arteries were not ligated. Eight groups (six rats in each group) were randomly assigned to a control group (CL), a stroke animal model (SAM) group, and three *H. erinaceus* wet mycelia groups (50, 300 and 1000 mg/kg), and three erinacine A groups (1, 5 and 10 mg/kg). Erinacine A was dissolved in DMSO and administered intraperitoneally during the 5 days before the onset of ischemia. *H. erinaceus* wet mycelia oral administration was indicated during the 5 days before ischemia.

### 4.3. Chemical Reagents and Antibodies

Mouse monoclonal antibodies against NeuN, GAPDH, β-actin, TNF-α, IL-1β, IL-6, iNOS and CHOP and rabbit polymonoclonal antibodies against nitrotyrosine were purchased from Santa Cruz Biotechnology (Santa Cruz, CA, USA). Rabbit monoclonal antibodies against phospho-p38 MAPK (Thr180/Tyr182) and phospho-JNK1/2 (Thr183/Tyr185) were purchased from Cell Signaling Technology (Beverly, MA, USA). The TdT-mediated dUTP Nick End Labeling (TUNEL) kits were purchased from Roche (Mannheim, Germany), SDS, NP-40, while sodium deoxycholate, protease inhibitor cocktail was purchased from Sigma (St. Louis, MO, USA).

### 4.4. Evaluation of the Size of the Ischemic Injury Using 2,3,5-Triphenyl tetrazolium chloride (TTC) Staining

The ischemic area was evaluated by TTC staining. Briefly, after the administration of a large dose of chloral hydrate, the rats were killed by decapitation after 24 h of reperfusion. The brains were quickly removed and placed in ice-cold saline for 10 min. The brains were then cut into seven 2 mm coronal slices using a rat brain matrix (Harvard Apparatus, Holliston, MA, USA). Sections were then incubated in 2% TTC (St. Louis, MO, USA) for 30 min and then immediately fixed in 10% formalin overnight. The infarcted area was outlined in white and was measured by image analysis software (Image-pro Plus medical image analysis, Media Cybernetics, Inc, Silver Spring, MD, USA) on the posterior surface of each section. The infarcted volume was calculated as the non-infarcted area of the ipsilateral hemisphere/total non-infarcted area (from both the ipsilateral and contralateral hemisphere) in order to avoid the influence of tissue edema. The mean volume of the brain infarction was calculated by summing the infarcted area in each slice multiplied by the thickness of the slice. The cortical infarcted volume was calculated by measuring cortical areas of infarction on a section-by-section basis, and the subcortical infarcted volume was equal to the difference between the total lesion volume and the cortical infarcted volume [37].

### 4.5. Immunohistochemistry

Immunohistochemistry (IHC) staining was performed using a biotinylated secondary antibody (Vectastain Universal Elite ABC Kit, Vector Laboratories, Inc., Burlingame, CA, USA). The omission of primary antibodies was used as the negative control. Using three slides, the presence of cytoplasm stained with brown was scored as positive. The expressions of NeuN, iNOS and nitrotyrosine were quantitatively evaluated using an Olympus Cx31 microscope with an Image-pro Plus medical image analysis system. The digital images were captured using a digital camera (Canon A640, Media Cybernetics, Inc, Silver Spring, MD, USA). The positive area and optical density of the positive cells were determined by measuring three randomly selected microscopic fields (400× magnification) on each slide. The image pixel index was defined as average integral optical density (AIOD) (AIOD = positive area × optical density/total area) [37].

### 4.6. Histopathological Evaluation

The brain tissue samples were fixed in 10% buffered formalin, embedded in paraffin and cut into 4 μm-thickness slides transversely from the neuron impairment area, for assessment by hematoxylin and eosin (H&E) staining. The histopathological changes in the neuron cells (NeuN, positive) were examined by BX51 light microscope (Olympus, Tokyo, Japan) at high power (200× magnification) for each slide. For quantitative purposes, both cortical and subcortical infarctions were blindly chosen from each slide, and photographs were then taken using an Image-pro Plus medical image analysis system. The cell numbers (NeuN, positive) were recorded. The control neurons were counted from ten fields randomly chosen from four groups (the sham group, the SAM group, and the six *H. erinaceus* groups) using a 200× magnification light microscope. The means of neuron cell numbers were calculated per microscope field from each of the six animals in each group [37].

### 4.7. Preparation of Total Cell Extracts and Immunoblotting Analysis

Cellular lysates were prepared by suspending brain tissue in 200 µL of lysis buffer (137 mM NaCl, 15 mM EGTA, 0.1 mM sodium orthovanadate, 15 mM MgCl_2_, 0.1% Triton X-100, 25 mM 3-(*N*-morpholino)propanesulfonic acid (MOPS), 100 µM phenylmethylsulfonyl fluoride and 20 µM leupeptin, adjusted to pH 7.2). The cells were then disrupted by sonication and extracted at 4 °C for 30 min. The protein content in the supernatant was quantified using a bicinchoninic acid assay (BCA assay), and then subjected to immunoblotting using Immobilon-P membranes (Millipore, Bedford, MA, USA) using the indicated secondary antibodies. Signals were detected using an enhanced chemiluminescence Western blot kit as described previously [38,39].

## 5. Conclusions

**Figure 7:**
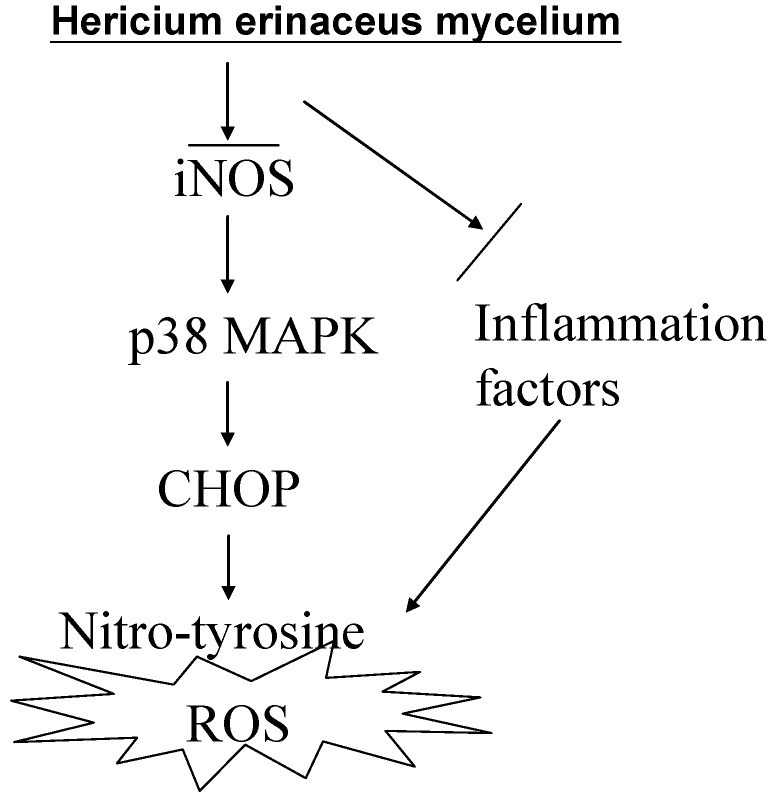
Schematic presentation of the signaling pathways involved in *H. erinaceus* mycelium inhibited transient global ischemia brain apoptosis and inflammation in rats. The effects of erinacine A on the scavenger of ROS, which inhibits iNOS, p38 MAPK and CHOP protein inactivation. These results suggest that the iNOS/p38 MAPK pathway may be involved in neuronal survival mediated by erinacine A after ischemic injury.

In conclusion, *H. erinaceus* offers neuroprotective effects after ischemic brain injury and has the ability to scavenge free radicals, this being related to endoplasmic reticulum stress signaling. It also inhibits inflammation by reducing the induction of iNOS and the production of nitrotyrosine. It does this via inactivation of p38/MAPK and reduction of the protein levels of CHOP. Taken together, our findings provide further insights into the mechanisms by which *H. erinaceus* exerts its beneficial effect on the injured brain (Figure 7). Finally, these findings suggest that erinacine A may be a promising agent that helps bring about neuroprotection and thus may reduce ischemic brain damage.
